# The use of honey in button battery ingestions: a systematic review

**DOI:** 10.3389/fped.2023.1259780

**Published:** 2023-09-28

**Authors:** Yannick Michael Schmidt, Oliver Muensterer, Danielle Wendling-Keim

**Affiliations:** Department of Pediatric Surgery, Dr. von Hauner Children's Hospital, University Hospital, LMU Munich, Munich, Germany

**Keywords:** honey, button battery, ingestion, esophagus, pediatric

## Abstract

**Background:**

Button battery (BB) ingestions may cause severe and possibly fatal complications, especially if the battery is located in the esophagus. The application of oral honey has recently been proposed by the National Capital Poison Center in the USA and in an ESPGHAN position paper in Europe, but clinical trials and experimental studies are limited. The goal of this systematic review was to analyze the evidence for this approach.

**Materials and methods:**

A systematic review of clinical trials and experimental studies on the oral application of honey after BB ingestion in children was performed. Inclusion criteria according to the PICO format were patient age 0–18 years, ingestion of BB, oral administration of honey or other substances, all *in vivo* and *in vitro* studies, as well as reported complication rate, esophageal injury, and mortality. A manual search in the databases MEDLINE, Web of Science and Cochrane was performed to identify relevant search terms to form the following queries and to construct the extensive search. Furthermore, the search was extended by using snowballing on the reports reference lists. The review is registered at Research Registry. The identifying number is reviewregistry1581.

**Results:**

We found four publications that investigated the effects of honey after button battery ingestion. Three of these presented experimental *in vitro* and *in vivo* results and one reported a clinical retrospective study of 8 patients.

**Conclusion:**

Follow up studies are required to further elucidate the effectiveness of the treatment with honey. The time intervals in which the use of honey is effective is not clear. Furthermore, a physiological model is needed for *in vitro* testing, preferably mimicking peristalsis and dynamic flow of the applied substances. However, since it is easy to apply and of minimal risk in patients over one year of age, honey should be considered a possible treatment option during the interval between presentation and endoscopic removal of the retained BB.

**Systematic Review Registration:**

https://www.researchregistry.com/browse-the-registry#registryofsystematicreviewsmeta-analyses/registryofsystematicreviewsmeta-analysesdetails/643e9df96750410027ee11b0/, identifier: reviewregistry1581.

## 1. Introduction

Button battery (BB) ingestion may lead to severe, sometimes fatal complications. Esophageal retention of a BB is associated with a particularly high rate of complications. The number of BB ingestions has been increasing rapidly throughout the past years following general technical advancement e.g., in the Unites States by 6.7 within 1985 through 2009, with almost two-thirds extracted from household devices by the patients (1, 2). Significant damage to the esophagus can occur as early as 2 h after ingestion, although *ex vivo* animal models showed macroscopically evident mucosal damage as early as 15 min after application of the BB (3). To minimize the associated risks, the European Society for Pediatric Gastroenterology Hepatology and Nutrition (ESPGHAN) formed a task force. In their position paper, they recommend the immediate localization of the battery and, if located in the esophagus, prompt removal within 2 h. If verification of the battery's localization is postponed >12 h, a computed tomography (CT)-scan is indicated to rule out possible vascular involvement (4). In an extensive metadata analysis, Varga et al. found BB ingestion associated with complications in 0.2%, with a mortality of 0.04%. Most complications affect the esophagus and can be subdivided in ulceration (22%), perforation (18%), trachea-esophageal fistula formation (15%), stricture/obstruction (14%), vascular involvement (6%), necrosis (5%), bilateral vocal cord palsy (2%), bronchopneumonia (0.4%), and spondylodiscitis (0.4%) (5). Patients under 6 years of age and ingestions of BB ≥ 2 cm in diameter bare the highest complication rate at 12% (1). The mechanism of injury is mainly due to pressure necrosis, electrical discharge, leakage of battery fluids and toxicity of the metal (5). Isothermal hydrolysis by the resulting alkaline solution causes alkaline injury to the surrounding tissue with colliquation necrosis (3).

A newly developed strategy to reduce any damage is the oral administration of honey during the interval between ingestion and retrieval of the battery. In 2018, Anfang et al. carried out *in vitro* experiments and *in vivo* animal trials regarding a possible protective effect of various substances including honey (6). As a result, the administration of honey has been implemented in the American guideline of the *National Capital Poison Center*. If button battery ingestion is suspected or confirmed in children older than 1 year of age [honey is associated with a risk of botulism in infants (7)] and the battery was swallowed less than 12 h ago, it recommends the administration of 10 ml honey every 10 min up to a total of 6 times (8). Further along the way, the European Society of Paediatric Gastroenterology, Hepatology and Nutrition issued a Position Paper which also recommends administering honey to children older than 1 year of age after BB ingestion (4). However, literature on the effects of honey after BB ingestion are rare, and these recommendations seem to be based on only three studies that have been published previously by Anfang et al. (6), by Gyawali et al. (9) and by Jia et al. (10). A small retrospective study points to a confirmation of the recommendation However, this study only included only 8 patients of whom only 2 received honey before button battery removal (11).

The objective of this systematic literature review was to identify all relevant literature regarding the use of honey in children (0–18 years) after esophageal button battery (BB) ingestion and analyze it regarding its potential protective effect. A structured literature search with evaluation of the resulting data was performed to establish whether the oral administration of honey after BB ingestion provides a benefit in the extent of esophageal injury, complication rate and mortality, compared to the administration of no or alternative substances.

## 2. Methods

We applied the *Preferred Reporting Items for Systematic Reviews and Meta-Analyses* (PRISMA) *2020 statement* standards and checklist for the literature review (12). The work has been reported in line with *Assessing the methodological quality of systematic reviews* (AMSTAR) Guidelines (13, 14). To minimize possible bias, a protocol was established before the search for suitable studies was initiated (see Supplementary Appendix) (15). MEDLINE (PubMed®), Web of Science and *Cochrane Central Register of Controlled Trials* (Central) were queried for literature on the use of honey in patients with potential or confirmed BB ingestion. Search terms included were the following: MEDLINE: (“Therapeutic Irrigation”[MeSH Terms] OR “Honey”[MeSH Terms] OR “Honey”[Text Word] OR “sirup”[Text Word] OR “glucos*”[Text Word] OR “sugar*”[Text Word] OR “therapeutic irrigat*”[Text Word]) AND (“electric power supplies/adverse effects”[MeSH Terms] OR “electric power supplies/methods”[MeSH Terms] OR “electric power supplies/trends”[MeSH Terms] OR “button batter*”[Text Word]). Web of Science: [ALL = (honey OR sirup OR glucos* OR sugar* OR “therapeutic irrigate*”)] AND ALL = (“button batter*”). Central: ([MeSH descriptor: (Honey) this term only] OR [MeSH descriptor: (therapeutic irrigation) this term only] OR Honey OR sirup OR glucos* OR “therapeutic irrigation” OR “therapeutic irrigations”) AND {[MeSH descriptor: (Electric power supplies) this term only] OR “button battery” OR “button batteries”}. All articles were reviewed independently by two investigators independently (YMS, DWK). Studies were reviewed in full-text detail when exclusion based on title/abstract was not possible. In- and exclusion criteria were strictly employed (see Supplementary Appendix). The research was conducted from February through September 2022.

## 3. Results

Our search strategy revealed three experimental and one clinical study assessing the use of honey in BB ingestions (see Tables 1, 2). In 2018, Anfang et al. investigated the administration of apple and orange juice, sports drinks, honey, maple syrup and sucralfate for a possible protective effect in case of BB ingestion. Initial *ex vivo* testing proofed honey and sucralfate to be neutralizing the batteries' effects. Subsequently, a transfer to porcine animal model verified the substances effect *in vivo*. Regarding optimal dosage and frequency, the authors followed physiological saliva production with appliance of 10 ml every 10–15 min (6). A second trial, published by Gyawali et al. in 2021 showed that BB previously covered with honey caused significantly less deep injury than an uncovered battery after 24 h in goat esophagi *ex vivo* (9). The third trial, published by Jia et al. in 2022 compared the effects of olive oil, honey and sucralfate *in vitro* and *in vivo* in a porcine model, finding honey and a mixture of honey and olive oil to reduce the injury to the tissue in comparison to the use of saline as a control (10).

**Table 1:** Overview of study design, setup and methods of three experimental studies (Anfang et al., Gyawali et al., Jia et al.)

Publication	The Laryngoscope, 2018			Indian J Otolaryngol Head Neck, Surg, 2021				Frontiers in Pediatric, 2022					
Compounds	Motts® apple juice			Pure honey				Olive oil					
	Orange juice (store brand)			Edible vinegar (pH 3,5; Acetic Acid)				Honey					
	Gatorade® (fruit punch, lemon-lime, berry blue)							Sucralfate					
	POWERADE® (mountain blast, fruit punch, lemon-lime)							Mixture honey + olive oil 1:1 (MOH)					
	Pure honey (8 different)												
	Pure maple syrup (store brand)												
	Carafate® (Sucralfate)												
	Simulated saliva												
Control	In vitro: 0.9% sodium chlorine, simulated saliva			Artificial saliva				In vitro: saline					
	In vivo: 0.9% sodium chlorine												
Battery	3 V lithium BB (3 V-CR2032)			3 V lithium BB (3 V-CR2032)				3 V lithium BB (3 V-CR2032)					
Setup	In vitro (*n* = 40)			In vitro (*n* = 40)				In vitro (*n* = 18)					
Model	Cadaveric porcine esophagi a 6 cm, longitudinal opened, Anode to mucosal surface			cadaveric goat esophagi (5 × 5 cm), Artificial saliva containing xylitol intermittently				cadaveric porcine esophagi a 5 cm					
Groups	20 compounds; each in duplicates			A (*n* = 10)	B (*n* = 10)	C (*n* = 10)	D (*n* = 10)	A (*n* = 3)	B (*n* = 3)	C (*n* = 3)	D (*n* = 3)	E (*n* = 3)	F (*n* = 3)
				BB only	BB coated w honey	BB only	BB only	Olive oil	Honey	Sucralfate	Olive oil	Honey	Sucralfate
				exposure up to 24 h	exposure up to 24 h	removed after 6 h	removed after 6 h	Irrigation every 10 min			Irrigation every 30 min		
						follow up 18 h	vinegar vs. artificial saliva						
							follow up 18 h						
Procedure	Incline 15°							Perpendicular to ground					
	*t* = 0 min: irrigation w saline							Irrigation with 10 ml every 10 min			Irrigation with 10 ml every 30 min		
	*t* = 10 min: wash w compound							for 2 h					
	*t* = 10–60 min: wash every 10–15 min w compound												
	*t* = 12 h: BB removed												
	In vivo (*n* = 9)							In vivo (*n* = 12)					
Model	American Yorkshire piglets 10–11 kg, Anode to posterior wall							Bama miniature piglets 9–12 kg, Anode to posterior wall,					
Groups	Sucralfate (1 g/10 ml)	Gunther's Pure Clover Honey	0.9% saline					Honey	Olive oil	MOH	Saline		
	(*n* = 3)	(*n* = 2)	(*n* = 4)					(*n* = 4)	(*n* = 4)	(*n* = 4)	(*n* = 4)		
Procedure	Incline 30°							Incline 30°					
** **	*t* = 0 min irrigation w saline							*t* = 0. min irrigation w 10 ml saline					
	*t* = 5 min wash w compound							*t* = 5 min irrigation w compound					
	*t* = 5–60 min: wash every 10 min w compound							*t* = 5–60 min: wash every 10 min w compound					
	t = 60 min: BB removed							*t* = 60 min: BB removed					
	*t* = 7 ± 0.5 days: esophagi removed after							*t* = 7 days: esophagi removed after					
Time of measurement	In vitro (all compounds)			In vitro (A vs. B; Honey)		In vitro (C vs. D; Vinegar)		In vitro (all compounds)					
Mucosal injury	12 h			30 min, 1, 3, 12, 24 h		6, 24 h		2 h					
pH	before placement, after removal			30 min, 1, 3, 12, 24 h		24 h		before placment, every 20 min for 2 h					
BB voltage	before placement, after removal							before placement, after removal					
Temperature change				Before placement, 1 h after removal									
	In vivo (Honey, Carafate®, Saline)							**In vivo (Honey, olive oil, MOH)**					
Mucosal injury	Removal of esophagi after 7 ± 0.5 days + 24 h in formalin							Removal of esophagi after 7 days					
pH	before placement, after removal												
BB voltage	before placement, after removal							before placement, after removal					
Method of measurement	In vitro			In vitro				In vitro					
Mucosal injury	Macroscopical inspection			Macroscopical/microscopipical inspection				Microscopical inspection, paraffin, 4 µm thickness, HE/eosin stain					
				0	no injury			Mucosal injury index score (MIIS) formed:					
				1	involvement of superficial mucosa only			0	no obvious lesions				
				2	involvement of partial muscle thickness			1	lesions + inflammatory cell infiltration in mucosal layer				
				3	involvement of complete muscle thickness			2	lesions + inflammatory cell infiltration in submucosal layer				
				4	involvement of outer serosa			3	lesions to muscular layer				
pH	Litmus paper			Litmus paper				indicator paper					
BB voltage	Voltmeter							voltmeter					
Temperature change				Infrared thermal gun									
	In vivo							In vivo					
Mucosal injury	Macroscopical	length						Macroscopical			Gross injury size		
** **		with of injury						Histological, 4 µm, HE/eosin stain			Depth of necrosis		
	Histological, 5 mm, HE stain	Depth of necrosis									Depth of granulation tissue		
		Depth of granulation tissue									Mucosal injury, MIIS		
		Muscular structure									Muscular injury length		
		Quality of injured and healed tissue											
BB voltage								Voltmeter					
Study design	In vitro: cadaveric animal model			In vitro: cadaveric animal model, blinded observer				In vitro: cadaveric animal model					
	In vivo: animal model							In vivo: animal model					
Statistics	One- / two-way analysis of variance with			Fisher's exact test, independent sample t-test				One-way analysis of variance, with Newman - Keul's test					
	*post hoc* Tukey correction using the calculated mean / standard deviation / number of subjects												

*(*p* < 0.05), **(*p* < 0.01), ***(*p* < 0.001), ****(*p* < 0.0001).

**Table 2:** Overview, categorization and comparison of collected results from three experimental studies (Anfang et al., Gyawali et al., Jia et al.).

	Anfang et al.							Gyawali et al.						Jia et al.				
pH	In vitro							In vitro						In vitro				
		30 min	90 min	120 min				time	w/o Honey	w Honey				Irrigation every 10 min	20 min	60 min	120 min	
	Saline (A) vs. Honey (B)	A > B (****)	A > B (****)	A > B (****)				30 min	9.5	8.5				Olive oil (A) vs. Honey (B)	A < B (**)	A < B (*)	n.s.	
	Saline (A) vs. Sucralfate (C)	A > C (****)	A > C (****)	A > C (****)				60 min	9.5	8.5				Olive oil (A) vs. Sucralfate (C)	A < C (**)	A < C (**)	A < C (*)	
	Honey (B) vs. Sucralfate (C)	B < C (n.s.)	B < C (**)	B < C (****)				3 h	11	8.5				Honey (B) vs. Sucralfate (C)	n.s.	B < C (**)	B < C (*)	
	Compound	ph 120 min	Neutralization Effectiveness	compound	ph 120 min	Neutralization Effectiveness		12 h	11	9				Irrigation every 30 min	20 min	60 min	120 min	
								24 h	11	9.25				Olive oil (D) vs. Honey (E)	D < E (**)	D < E (*)	n.s.	
	Honey			POWERADE®				t-test	***					Olive oil (D) vs. Sucralfate (F)	D < F (**)	D < F (**)	D < F (*)	
	Madhava Very Raw	4.5	Ideal	mountain blast	10.5	Minimal, no benefit								Honey (E) vs. Sucralfate (F)	n.s.	E < F (*)	E < F (*)	
	Makuna Bio Active	4.8	Ideal	fruit punch	11.5	Minimal, no benefit												
	Raw Organic Honey	5	Ideal	lemon-lime	11.5	Minimal, no benefit												
	Buzz & Bloom Bold	5.5	Ideal	Gatorade®														
	Crockett Arizona Wildflower	5.5	Ideal	fruit punch	11	Minimal, no benefit												
	Gunter's Honey Clover	6	Ideal	lemon-lime	11.5	Minimal, no benefit												
	Linden Smiley Unfiltered	6	Ideal	berry blue	12	Minimal, no benefit												
	Nature Nate's Raw & Unfiltered	7.5	Ideal	maple syrup	11.5	Minimal, no benefit												
	Carafate® (Sucralfate)	7.5	Ideal	Control														
	Juice			Simulated saliva	12.8	No benefit												
	Motts® apple juice	9	Partial	0.9% sodium chloride	13	No benefit												
	orange juice	9.5	Partial															
	In vivo																	
		60 min																
	Saline (A) vs. Honey (B)	A > B (**)																
	Saline (A) vs. Sucralfate (C)	A > C (*)																
Mucosal injury	In vitro							In vitro						In vitro				
		120 min						time	degree	w/o Honey	w Honey				Olive oil every 10 min (A)	Olive oil every 30 min (D)		
	Saline (A) vs. Honey (B)	A > B						30 min	0	0	3							
	Saline (A) vs. Sucralfate (C)	A > C							1	10	7			vs. Honey (B)	A < B (*)	n.s.		
									2	0	0			vs. Sucralfate (C)	A < C (**)	n.s.		
									3	0	0			vs. Honey (E)	A < E (**)	D < E (*)		
									4	0	0			vs. Sucralfate (F)	A < F (**)	D < F (*)		
	In vivo							60 min	0	0	1			In vivo				
	macroscopically	ulcer area	severity	perforation					1	7	9			macroscopically	ulcer size			
	Saline (A)			2 (4) 50%					2	3	0			Saline (A) vs. Olive oil (B)	n.s.			
	Saline (A) vs. Honey (B)	n.s.	B < A	0 (2) 0%					3	0	0			Saline (A) vs. Honey (C)	A > C (**)			
	Saline (A) vs. Sucralfate (C)	n.s.	C < A	0 (3) 0%					4	0	0			Saline (A) vs. MOH (D)	A > D (**)			
	histologically	depth of nectrotic tissue	depth of granualtion tissue	extension of muscular injury beyond surface ulcer	number of breaks in muscularis propria	distance of breaks in muscularis propria	distance per break in muscularis propria	3 h	0	0	1			Honey (C) vs.Olive oil (B)	n.s.			
									1	7	9			Honey (C) vs. MOH (D)	C > D (*)			
	Saline (A) vs. Honey (B)	A > B (*)	A > B (*)	A > B (*)	A > B (*)	A > B (*)	n.s.		2	3	0			histologically	Depth of necrotic tissue	Depth of granulation tissue	Mucosal injury	Muscular injury
	Saline (A) vs. Sucralfate (C)	A > C (*)	A > C (*)	A > C (*)	A > C (*)	A > C (*)	n.s.		3	0	0							
	Subgroups macroscopically and histologically		gross size of ulcer	extension of muscular injury beyond surface ulcer					4	0	0							
								12 h	0	0	0			Saline (A) vs. Olive oil (B)	A < B (**)	n.s.	n.s.	n.s.
	Perforated saline (A-PC) vs. Honey (B)		A-PC > B (*)	n.s.					1	0	0			Saline (A) vs. Honey (C)	A > C (**)	A > C (*)	n.s.	n.s.
	Perforated saline (A-PC) vs. Sucralfate (C)		A-PC > C (*)	n.s.					2	4	9			Saline (A) vs. MOH (D)	A > D (**)	A > D (**)	A > D (**)	A > D (**)
	Non-perforated Saline (A-NPC) vs. Honey (B)		n.s.	A-NPC > B (*)					3	6	1			Honey (C) vs.Olive oil (B)	n.s.	n.s.	n.s.	n.s.
	Non-perforated Saline (A-NPC) vs. Sucralfate (C)		n.s.	A-NPC > C (*)					4	0	0			Honey (C) vs. MOH (D)	n.s.	C > D (**)	n.s.	C > D (**)
	Perforated saline (A-PC) vs. Non-perforated Saline (A-NPC)		A-PC > A-NPC (*)	A-PC < A-NPC (*)				24 h	0	0	0							
									1	0	0							
									2	0	3							
									3	2	7							
									4	8	0							
Temperatur								In vitro										
											w/o Honey	w Honey	t-test					
								before placement (°F)			73.97	73.98	***					
								after 1 h (F°)			77.5	77.6						
								delta temperature (°F)			3.53	3.62						
BB voltage	In vivo													In vitro				
		delta V 60 min													residual V	delta V		
	Saline (A) vs. Honey (B)	A > B (**)												Olive oil (A) vs. Honey (B)	A > B (**)	A < B (**)		
	Saline (A) vs. Sucralfate (C)	A > C (*)												Olive oil (A) vs. Sucralfate (C)	A > C (**)	A < C (**)		
														Olive oil (A) vs. Olive oil (D)	A > D (**)	A < D (*)		
														Olive oil (A) vs. Honey (E)	A > E (**)	A < E (**)		
														Olive oil (A) vs. Sucralfate (F)	A > F (**)	A < F (**)		
														Honey (B) vs. Sucralfate (C)	B > C (**)	B < C (**)		
														Honey (B) vs. Olive oil (D)	n.s.	n.s.		
														Honey (B) vs. Honey (E)	B > E (**)	B < E (**)		
														Honey (B) vs. Sucralfate (F)	B > F (**)	B < F (**)		
														In vivo				
															residual V	delta V		
														Saline (A) vs. Olive oil (B)	n.s.	n.s.		
														Saline (A) vs. Honey (C)	A < C (*)	n.s.		
														Saline (A) vs. MOH (D)	A < D (**)	A > D (**)		
														Honey (C) vs.Olive oil (B)	n.s.	n.s.		
														Honey (C) vs. MOH (D)	C < D (**)	C > D (**)		

*(*p* < 0.05), **(*p* < 0.01), ***(*p* < 0.001), ****(*p* < 0.0001).

The extend of tissue injury was measured *in vitro* in all three experimental studies with Anfang et al. and Gyawali et al. showing less damage to the tissue when applying honey compared to untreated or with saline treated tissue. Anfang et al. were able to demonstrate a protective effect for sucralfate as well. Depending on the interval of the irrigations, Jia et al. were able to show an equivalent or partly greater protective effect for the use of olive oil, compared to honey or sucralfate. However, all three experimental studies reported qualitative results only. Anfang et al. compared the extent and Gyawali et al. and Jia et al. evaluated the injury with their self-defined ordinal scale. To date, there are only two experimental studies assessing the *in vivo* effects of irrigations with honey in case of BB ingestion. In both studies, treatment with honey led to less injury of the esophagus with smaller ulcer size. There was no peroration when honey or sucralfate was used, in contrast to the piglets which were treated with saline only and developed perforation in 50% of the cases (6).

The histopathological examination of the esophagi as well revealed weaker extent of the BB induced damage when treated with honey or sucralfate (6) respectively honey or a mixture of honey and olive oil (MOH) (10). According to the authors, honey reduced the depth of the necrosis, the depth of granulation tissue, as well as the muscular injury induced by the BB. With regards to the *in vitro* results, Jia et al. saw perforation in all piglets treated with olive oil alone. This group also reported more favorable *in vivo* tissue protecting effects when using MOH rather than using honey alone.

The effects of honey on temperature in the affected area as another factor was examined by Gyawali et al. and showed no clinically relevant difference.

Anfang et al. and Jia et al. assessed the effects of their substances on the change of voltage within the used BB. *In vivo* they found honey and sucralfate to reduce the change in voltage across tissue (6), and honey and a MOH to reduce the loss of voltage, respectively the change of voltage when compared to saline.

The fourth study that we found was retrospective in nature and included 8 patients. The time to battery removal as well as the battery size varied, but the age was quite uniform between 1 and 3 years. Patients who were treated with honey did not develop any complications. These patients also received acetic acid after removal and had a shorter time to removal than most of the other patients so that we are facing a potential bias. No adverse effects of the application of honey after button battery ingestion were seen in this study (11).

## 4. Discussion

To our knowledge, this is the first systematic analysis of studies on the subject of applying honey to mitigate the effects of retained esophageal BB. Surprisingly, without much underlying data, this type of adjuvant therapy has made its way into guidelines on both sides of the Atlantic.

According to our review, the use of honey may be protective, not only by neutralizing the battery induced pH change, but also by forming a shielding film around the BB due to its higher viscosity.

Contrary to the assumption that exothermic neutralization could induce relevant thermal damage, no evidence of such was found and the observed rise in temperature in animal models was limited to 0–3°C (3, 16).

The results of the Anfang et al. trial have already been implemented into the recommendations of the *National Capital Poison Center* (United States), which advises the administration of honey in suspected or confirmed BB ingestion in children >1 year of age [risk of botulism in younger children, see above (7)] and swallowing of the battery <12 h ago. According to their recommendations, 10 ml of honey should be given orally every 10 min until recovery of the foreign body and maximum 6 times (8). Since esophageal perforation in BB ingestion is rare within the first 12 h (<2% of all perforations), potential adverse events should be negligible and administration of honey in the initial period therefore can be considered safe (17). The potentially increased risk of aspiration at induction of anesthesia due to oral intake (2.2/100.000 non-elective procedures) is neglectable in light of the small volume of honey ingested and in comparison, to the risk of a rapidly progressing, potentially fatal injury to the esophagus (18, 19).

The inclusion and exclusion criteria of our study led to a limited number of only four studies. However, the number of *in vitro* experiments in each study was sufficient to provide a basic knowledge of the time from BB ingestion to different extents of damage of the esophagus as well as the effects of various agents on the pH. In addition, the potential danger from high temperature seems to be ruled out. Nevertheless, the number of microscopic studies as well as *in vivo* studies was limited to a number *n* = 9 (Anfang et al.) respectively *n* = 12 (Jia et al.), and the application of honey was only investigated twice histologically and *in vivo*.

Therefore, prospective follow up studies are required to further elucidate the effectiveness of the treatment with honey or sucralfate. Furthermore, the time intervals in which the use of honey or sucralfate is effective is not clear since the studies applied the honey immediately with or even before the battery, which is not realistic in the clinical setting and the studies lack of testing a wide variety of potential intervals. Likewise, a more physiological model is needed for the *in vitro* testing, possibly mimicking the peristalsis and the flow of the applied substances which was lacking throughout the *in vitro* settings of the described studies. In addition, performance of clinical trials with a larger number of participants are needed in the future.

Due to heterogeneity of those studies in approach, setup, and analysis, the performance of a meta-synthesis or -analysis was only limited and descriptively possible. Nevertheless, these studies have provided a basic insight into the effects of honey after BB ingestion. In detail, the studies investigated the pH change, the extent of the mucosal injury, the temperature, and the voltage (see Tables 1, 2). *In vitro* the pH was decreased by honey more than by saline or sucralfate, and this effect increased over time. Furthermore, the pH was decreased by sucralfate more than by saline. Jia et al. also assessed the *in vitro* effect of olive oil, which lowered the pH more than honey or sucralfate. Neutralization effectiveness of honey and sucralfate after 120 min was ideal, whereas fruit juices and various Sports drinks as well as saliva and physiological sodium chloride solution did not recover the pH measured on the mucosa. *In vivo* pH testing was performed only by Anfang et al. and confirmed said observations with honey and sucralfate decreasing the pH more than saline did.

Our systematic review has several limitations. Due to heterogeneity of the assessed studies, a direct comparison of their results is limited. A statistical evaluation was therefore not reasonable and not performed. Studies published only in small databases might have been missed.

The most important limitation at this time is the lack of clinical comparative studies. These studies are difficult to perform, because the event incidence is low. Therefore, we are currently preparing a multicenter, prospective trial to objectively test the effect of honey in a systematic fashion. Nevertheless, the available studies suggest a positive effect, with minimal risks and disadvantages for the patients. Therefore, at this time, administering honey after suspected button battery injestion is advisable.

Our findings indicate that when BB ingestion is suspected or confirmed, a coordinated rapid approach to minimize the risk of complications is needed (20) and the oral administration of honey in the interval between ingestion and retrieval could potentially reduce complications. This approach should not delay the removal of the battery (1, 21).

In an experimental study that currently carried out at our clinic, we are addressing the above-mentioned weaknesses of previous studies. We are including, among other things, various battery types, as well as different application intervals and types of honey with different viscosities.

With only three experimental and one clinical study available so far, there is a great need for a large, prospective, and ideally multicenter study to carefully evaluate the effect of honey used in children with BB ingestion. This is the only way to assess whether the mentioned measures are not only safe, but also effective.

### Other information/limitations

The review is registered at Research Registry. The identifying number is reviewregistry1581.

By limiting our research to only English publications, the possibility of a publication bias given.

## Data Availability

The original contributions presented in the study are included in the article/**Supplementary Material**, further inquiries can be directed to the corresponding author.

## References

[1] Litovitz T Whitaker N Clark L White NC Marsolek M. Emerging battery-ingestion hazard: clinical implications. Pediatrics. (2010) 125(6):1168–77. 10.1542/peds.2009-3037 20498173

[2] Litovitz T Whitaker N Clark L. Preventing battery ingestions: an analysis of 8648 cases. Pediatrics. (2010) 125(6):1178–83. 10.1542/peds.2009-3038 20498172

[3] Jatana KR Rhoades K Milkovich S Jacobs IN. Basic mechanism of button battery ingestion injuries and novel mitigation strategies after diagnosis and removal. Laryngoscope. (2017) 127(6):1276–82. 10.1002/lary.26362 27859311

[4] Mubarak A Benninga MA Broekaert I Dolinsek J Homan M Mas E Diagnosis, management, and prevention of button battery ingestion in childhood: a European society for paediatric gastroenterology hepatology and nutrition position paper. J Pediatr Gastroenterol Nutr. (2021) 73(1):129–36. 10.1097/MPG.0000000000003048 33555169

[5] Varga Á Kovács T Saxena AK. Analysis of complications after button battery ingestion in children. Pediatr Emerg Care. (2018) 34(6):443–6. 10.1097/PEC.0000000000001413 29369262

[6] Anfang RR Jatana KR Linn RL Rhoades K Fry J Jacobs IN. pH-neutralizing esophageal irrigations as a novel mitigation strategy for button battery injury. Laryngoscope. (2019) 129(1):49–57. 10.1002/lary.27312 29889306

[7] Midura TF. Update: infant botulism. Clin Microbiol Rev. (1996) 9(2):119–25. 10.1128/CMR.9.2.119 8964030 PMC172885

[8] Center NCP. Button battery ingestion triage and treatment guideline. 2010–2022. Available at: https://wwwpoisonorg/battery/guideline

[9] Gyawali BR Guragain R Gyawali DR. Role of honey and acetic acid in mitigating the effects of button battery in esophageal mucosa: a cadaveric animal model experimental study. Indian J Otolaryngol Head Neck Surg. (2022) 74(Suppl 3):5759–65. 10.1007/s12070-021-02382-6 36742683 PMC9895233

[10] Jia W Xu G Xie J Zhen L Chen M He C Electric insulating irrigations mitigates esophageal injury caused by button battery ingestion. Front Pediatr. (2022) 10:804669. 10.3389/fped.2022.804669 35633974 PMC9133442

[11] Chandran D Park S Barker R Burns H. Management of oesophageal impaction of button batteries in Queensland. ANZ J Surg. (2022) 92(9):2115–22. 10.1111/ans.17638 35373432

[12] Page MJ McKenzie JE Bossuyt PM Boutron I Hoffmann TC Mulrow CD The PRISMA 2020 statement: an updated guideline for reporting systematic reviews. Br Med J. (2021) 372:n71. 10.1136/bmj.n71 33782057 PMC8005924

[13] Shea BJ Grimshaw JM Wells GA Boers M Andersson N Hamel C Development of AMSTAR: a measurement tool to assess the methodological quality of systematic reviews. BMC Med Res Methodol. (2007) 7:10. 10.1186/1471-2288-7-10 17302989 PMC1810543

[14] Shea BJ Reeves BC Wells G Thuku M Hamel C Moran J AMSTAR 2: a critical appraisal tool for systematic reviews that include randomised or non-randomised studies of healthcare interventions, or both. Br Med J. (2017) 358:j4008. 10.1136/bmj.j4008 28935701 PMC5833365

[15] Shamseer L Moher D Clarke M Ghersi D Liberati A Petticrew M Preferred reporting items for systematic review and meta-analysis protocols (PRISMA-P) 2015: elaboration and explanation. BMJ. (2015) 349:g7647. 10.1136/bmj.g7647 25555855

[16] Jatana KR Barron CL Jacobs IN. Initial clinical application of tissue pH neutralization after esophageal button battery removal in children. Laryngoscope. (2019) 129(8):1772–6. 10.1002/lary.27904 30835848

[17] Soto PH Reid NE Litovitz TL. Time to perforation for button batteries lodged in the esophagus. Am J Emerg Med. (2019) 37(5):805–9. 10.1016/j.ajem.2018.07.035 30054113

[18] Hoagland MA Ing RJ Jatana KR Jacobs IN Chatterjee D. Anesthetic implications of the new guidelines for button battery ingestion in children. Anesth Analg. (2020) 130(3):665–72. 10.1213/ANE.0000000000004029 30829672

[19] Walker RW. Pulmonary aspiration in pediatric anesthetic practice in the UK: a prospective survey of specialist pediatric centers over a one-year period. Paediatr Anaesth. (2013) 23(8):702–11. 10.1111/pan.12207 23763657

[20] Russell RT Griffin RL Weinstein E Billmire DF. Esophageal button battery ingestions: decreasing time to operative intervention by level I trauma activation. J Pediatr Surg. (2014) 49(9):1360–2. 10.1016/j.jpedsurg.2014.01.050 25148737

[21] Sethia R Gibbs H Jacobs IN Reilly JS Rhoades K Jatana KR. Current management of button battery injuries. Laryngoscope Investig Otolaryngol. (2021) 6(3):549–63. 10.1002/lio2.535 34195377 PMC8223456

