# Supplementary material for: The use of honey in button battery ingestions: a systematic review
Source: Front Pediatr. 2023 Sep 28;11:1259780. doi: 10.3389/fped.2023.1259780 (PMC10569471; doi:10.3389/fped.2023.1259780)
Supplement: Supplementary file 2 [file Datasheet1.docx]

**Appendix**

**Protocol**

Objective

This systematic review aims to find data to support the use of orally administered honey in children with suspected or confirmed button battery (BB) ingestion.

Inclusion/exclusion criteria

The inclusion/exclusion criteria were determined using PICO process (1).

|  | Inclusion criteria | Exclusion criteria |
| --- | --- | --- |
| **P**opulation | - Patients age 0-18 years - Ingestion of BB - In vivo / in vitro studies | - Patients age >18 years |
| **I**ntervention | - Oral administration of honey |  |
| **C**omparison | - Oral administration of other or no substances |  |
| **O**utcomes | - Complication rate - Esophageal injury - Mortality |  |

This systematic review considered all studies evaluating oral administration of honey in children with suspected or confirmed BB ingestion and no study type was excluded.

Databases

MEDLINE, Web of Science, Cochrane Central Register of Controlled Trials (Central)

Search strategy

A manual search in the databases mentioned above was performed to identify relevant search terms to form the following queries to construct the extensive search.

MEDLINE:

("Therapeutic Irrigation"[MeSH Terms] OR "Honey"[MeSH Terms] OR "Honey"[Text Word] OR "sirup"[Text Word] OR "glucos*"[Text Word] OR "sugar*"[Text Word] OR "therapeutic irrigat*"[Text Word]) AND ("electric power supplies/adverse effects"[MeSH Terms] OR "electric power supplies/methods"[MeSH Terms] OR "electric power supplies/trends"[MeSH Terms] OR "button batter*"[Text Word])

Web of Science:

(ALL=(honey OR sirup OR glucos* OR sugar* OR “therapeutic irrigate*”)) AND ALL = (“button batter*“)

Central:

((MeSH descriptor: [Honey] this term only) OR (MeSH descriptor: [therapeutic irrigation] this term only) OR Honey OR sirup OR glucos* OR „therapeutic irrigation“ OR „therapeutic irrigations“) AND ((MeSH descriptor: [Electric power supplies] this term only) OR „button battery“ OR „button batteries“)

Furthermore, the search was extended by using snowballing on the reports reference lists.

Critical appraisal

Detailed review was performed including full text and studies were included if consent was found by two reviewers (YMS, DWK). A third reviewer would have mediated in case of disagreement of the two reviewers (2).

Methodology data collection/synthesis

See Flow Chart

Data statement

No new data was collected. All data in this publication is obtained from the cited original publications.

1. Schardt C, Adams MB, Owens T, Keitz S, Fontelo P. Utilization of the PICO framework to improve searching PubMed for clinical questions. BMC medical informatics and decision making. 2007;7:16-.

2. van Tulder M, Furlan A, Bombardier C, Bouter L. Updated method guidelines for systematic reviews in the cochrane collaboration back review group. Spine (Phila Pa 1976). 2003;28(12):1290-9.
