# Supplementary material for: The use of honey in button battery ingestions: a systematic review
Source: Front Pediatr. 2023 Sep 28;11:1259780. doi: 10.3389/fped.2023.1259780 (PMC10569471; doi:10.3389/fped.2023.1259780)
Supplement: Supplementary file 4 [file Image1.pdf]

PRISMA 2009 Flow Diagram

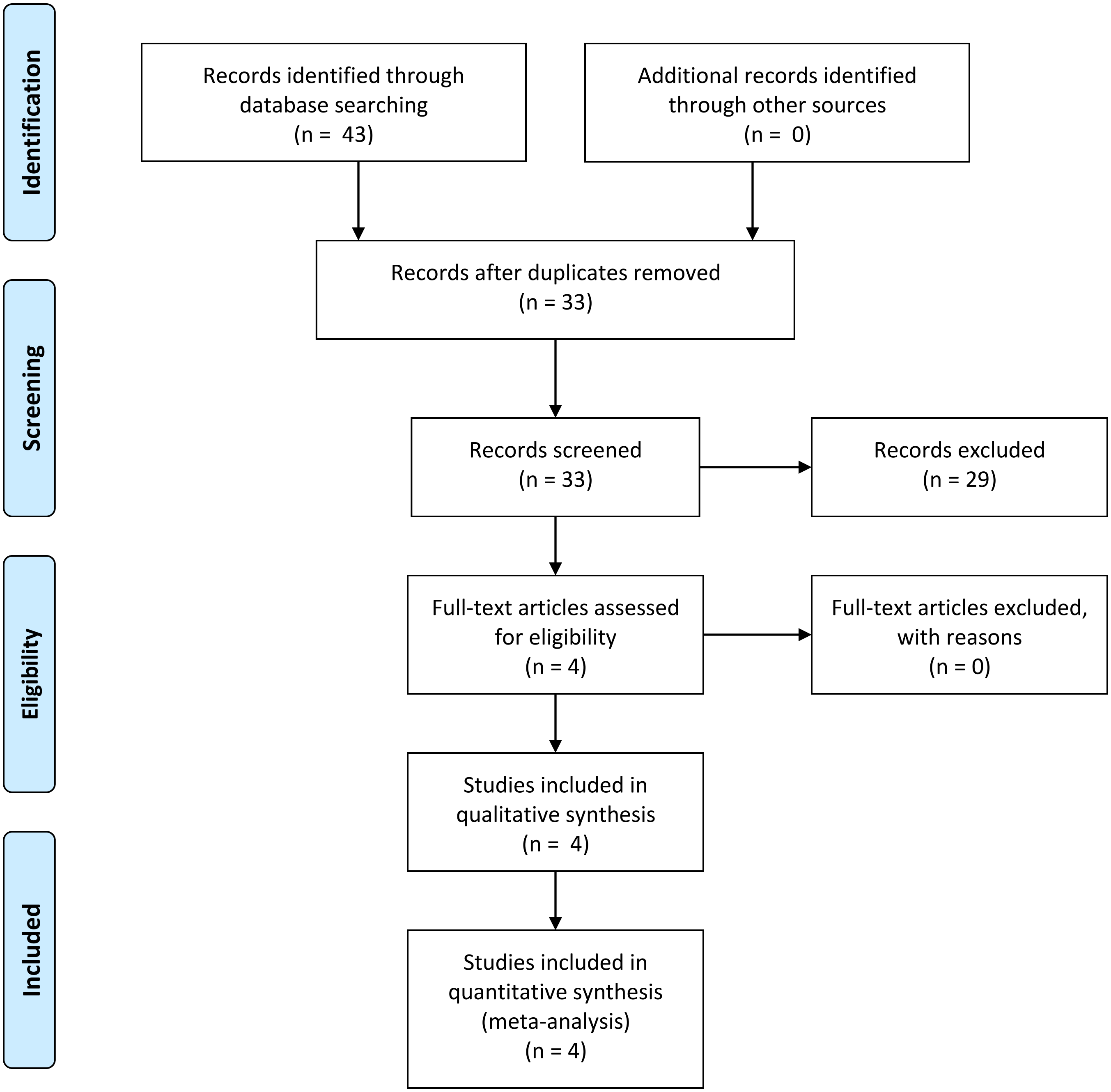

From: Moher D, Liberati A, Tetzlaff J, Altman DG, The PRISMA Group (2009). *P*referred *R*eporting *I*tems for *S*ystematic Reviews and *M*eta-*A*nalyses: The PRISMA Statement. PLoS Med 6(7): e1000097. doi:10.1371/journal.pmed1000097

For more information, visit www.prisma-statement.org.
